# Evaluation of the quality and value of data sources for postmarket surveillance of the safety of cough and cold medications in children

**DOI:** 10.1186/s12874-018-0626-3

**Published:** 2018-12-22

**Authors:** Jody L. Green, Kate M. Reynolds, William Banner, G. Randall Bond, Ralph E. Kauffman, Robert B. Palmer, Ian M. Paul, Richard C. Dart

**Affiliations:** 1 Rocky Mountain Poison & Drug Center, Denver Health and Hospital Authority, 1391 Speer Blvd, 777 Bannock St. MC 0180, Denver, CO 80204 USA; 2 Oklahoma Center for Poison and Drug Information, Oklahoma University College of Pharmacy, 940 NE 13TH. St, Oklahoma City, OK 73104 USA; 3 Faculty of Medicine, Hope Africa University, Avenue Buconyori, Ngagara Q2, Bujumbura, Burundi; 4 Department of Pediatrics, University of Missouri-Kansas City School of Medicine, 2301 Holmes St, Kansas City, MO 64108 USA; 5 Pediatrics & Public Health Sciences, Penn State College of Medicine, 700 HMC Crescent Rd, Hershey, PA 17033 USA; 6 Inflexxion Inc, 890 Winter St, Suite 235, Waltham, MA 02451 USA

**Keywords:** Adverse drug reaction reporting systems, Cold and flu medications, Data quality, Drug safety, Nonprescription drugs, Postmarket drug surveillance

## Abstract

**Background:**

The purpose of this report is to evaluate the quality of data sources used to study cough and cold medication (CCM) safety in children via the Pediatric Cough and Cold Safety Surveillance System.

**Methods:**

The System utilized the National Poison Data System (NPDS), FDA Adverse Event Reporting System (FAERS), English-language medical literature, manufacturer postmarket safety databases, and news/media reports to identify cases from January 2008 through September 2016. Each data source was evaluated by the proportion of *detected* cases determined to be *eligible* (met case criteria) and the proportion determined to be *evaluable* (able to determine causal relationship between adverse event and exposure).

**Results:**

A total of 7184 unique cases were identified from 27,597 detected reports. Of these, 6447 (89.7%) were evaluable. The data source with the highest volume of detected cases was news/media; however, only 0.3% of those cases were eligible for panel review and only 0.2% (24 out of 13,450 cases) were evaluable. The data source with the highest proportion of eligible and evaluable cases was NPDS with 7691 detected cases, 6113 (79.5%) eligible cases, and 5587 (72.6%) evaluable cases.

**Conclusions:**

The data sources utilized to evaluate the safety profile of pediatric CCMs yielded variable detection and evaluation rates, but overall provided a comprehensive look at exposures that otherwise cannot be studied in clinical trials. While this study suggests that each source made a valuable contribution and that evaluable cases are generalizable, improvements are needed in case completeness and accuracy to enhance the quality of postmarket safety evaluations.

**Electronic supplementary material:**

The online version of this article (10.1186/s12874-018-0626-3) contains supplementary material, which is available to authorized users.

## Background

Postmarket surveillance of products approved by the United States Food and Drug Administration (FDA) provides real-world patient and provider experience and allows for monitoring of the product outside the confines and protections of randomized controlled clinical trials. In most instances, postmarket experience gives patients and providers confidence when expected safety profiles are supported with real-world data. However, this type of surveillance has played a critical role in discovering safety signals that were not detected pre-market and in extreme cases resulted in removal of unsafe prescription products from the market. A couple of well publicized examples of drug removal include that of rofecoxib [1] and Opana®.[2] While postmarket surveillance plays a critical role in patient safety, the methods by which data are obtained, characterized, and reported, as well as the quality of the data sources, are not well-studied.

The majority of cough and cold medications (CCMs) are “monograph drugs” meaning that they were marketed prior to May 1972 at which time a public rule-making process determined standards for safety and efficacy [3]. Examples of monograph drugs are products containing acetaminophen, diphenhydramine, and aspirin, all of which are common medications found in today’s household medicine cabinets [4]. The available evidence regarding the efficacy and safety of an over-the-counter (OTC) monograph drug is often sparse, particularly for children, due to the historical nature of products that have been readily available for decades and deemed as “generally recognized as safe and effective” by the FDA [5, 6].

One significant challenge to understanding the safety profile of these and other OTC medications is the collection of relevant real-world data. Randomized controlled clinical trials require large, hard-to-reach samples with limited potential to detect unintended exposures, dosing errors, and other real-world experiences. These types of exposures are essential to completing the safety profile. As previously described [7–9], a “mosaic” approach mitigates limitations of epidemiological designs by using diverse data sources to overcome weaknesses of individual sources, thereby increasing the reliability and validity of results. The approach has been used in many fields of research and is especially useful in difficult-to-study patient populations, such as children exposed to CCMs. Real-world events such as accidental unsupervised ingestions or medication errors are typically difficult to study prospectively due to the relative infrequency and unpredictable nature of such events. Hence, randomized controlled trials are of limited value. Instead, researchers must rely on multiple convenience samples. No single data source is expected to provide complete and representative information but when considered together, multiple data sources may strengthen the credibility of findings, reduce the risk of false interpretations, and provide a more complete and comprehensive perspective on the behaviors of the group.

With the mosaic approach in mind, an active postmarket surveillance system was developed in 2008 to monitor pediatric exposures to CCMs as reported to multiple data sources. The Pediatric Cough and Cold Safety Surveillance System detects and gathers cases of clinical events associated with pediatric exposures to CCMs from multiple data sources on a continuous basis. The objectives of this ongoing surveillance system are to conduct safety surveillance through monitoring of adverse event cases of oral nonprescription CCM exposures in children < 12 years old and to perform a root cause analysis to characterize the risk factors. The purpose of this report is to evaluate the quality and value of the data sources utilized in the Pediatric Cough and Cold Safety Surveillance System.

## Methods

The data sources utilized in the Pediatric Cough and Cold Safety Surveillance System include the National Poison Data System (NPDS), the FDA Adverse Event Reporting System (FAERS), English-language medical literature, postmarket safety databases of participating manufacturers, and news/media reports. Case inclusion criteria from each data source were: patient less than 12 years of age; exposure to at least one product that contains one or more of the eight most common active pharmaceutical ingredients (APIs) in CCMs (brompheniramine, chlorpheniramine, dextromethorphan, diphenhydramine, doxylamine, guaifenesin, pseudoephedrine, and phenylephrine); report of at least one significant adverse event as defined by the case definition for each data source (Table 1); and the event occurred in the United States. Each data source had its own case definition, identification, and acquisition process due to the variations of standard data fields, data structure, and accessible information between data sources (Table 1; Additional file 1) [10]. Autopsy reports were sought as supplemental information for each fatality and were included with the source information for a case when available. Details of the methods employed have been previously described [10].

**Table 1:** Data Source Inclusion Criteria for Eligibility and Definitions of Relatedness for Evaluability

Data Source (Eligible)	
National Poison Data System (NPDS)	• NPDS is the data repository of records of exposures to pharmaceutical and non-pharmaceutical substances reported to all regional poison centers located throughout the United States. • Cases were defined as those that reported a medical outcome of moderate effect, major effect, or death.
Food and Drug Administration Adverse Event Reporting System (FAERS)	• FAERS is the database that contains adverse event reports, medication error reports and product quality complaints resulting in adverse events that were submitted to FDA. • Fatal and non-fatal serious adverse events based upon the International Conference on Harmonisation (ICH) definition
English Language Medical Literature	• Published scientific reports in abstract or full paper form. • Cases were defined as those that met minimum eligibility criteria.
Participating Manufacturer Postmarket Safety Databases	• Medication error or accident records reported to drug manufacturers via postmarket surveillance requirements • Fatal and non-fatal serious adverse events based upon the International Conference on Harmonisation (ICH) definition
News/Media Reports	• News and media reports indexed from an aggregate database of > 32,000 sources of headline information • Cases were defined as those that met minimum eligibility criteria.
Definitions of Relatedness (Evaluable)^**a**^	
Related	• History of ingestion consistent with exposure • Drug levels consistent with exposure, if available • Clinical course consistent with exposure • No other cause of death/event evident
Potentially Related	• History of ingestion consistent with exposure • Drug levels consistent with exposure, if available • Clinical course consistent with exposure • Other cause of death/event unlikely • Drug may have been secondary cause of death/event
Unlikely Related	• No history of ingestion • Drug levels inconsistent with exposure • Clinical course inconsistent with exposure • Other cause of death/event possible
Unable to Determine if Related	• Not enough case detail to evaluate relationship of drug to death/event

^a^The definitions of each relatedness category include the general characteristics of that definition. Cases meeting that definition are not required to have all defined characteristics

Cases collected through each data source are entered and tracked through a central database to merge all findings. A systematic process of both electronic database algorithms and manual review is used to identify duplicate cases based upon the established Guideline on Detection and Management of Duplicate Individual Cases and Individual Case Safety Reports (ICSRs) published by the European Medicines Agency [11]. Age and gender, date of event, products reported, concomitant medications, comorbid conditions, reported events, drug concentration levels, scenario, and outcome are used to identify duplicate reports. All source data for duplicate cases are combined to compile one unique case record for panel review, preserving all information related to the case (Additional file 1).

A standardized case report form is used to collect key data elements, regardless of data source. The case report form captures patient demographics (age, gender, weight), exposure reason, exposure characteristics, substances involved (organized by active pharmaceutical ingredient), dose and duration of exposure, clinical course including laboratory values and other temporally associated indicators, adverse event terms (standardized by Medical Dictionary for Regulatory Activities (MedDRA)), version 19.0 (MSSO, Chantilly, Virginia), drug concentrations, and outcome (death: yes/no). Case report forms and all case source documents are distributed to the Pediatric Cough and Cold Surveillance System Consensus Panel members prior to each Panel review meeting. While all parameters were not present in each case, every effort was made to include all available information on each case.

The Panel membership consisted of five experts from the specialties of pediatrics, clinical pharmacology, toxicology, intensive care, emergency medicine, and forensic toxicology. The same members were active through the duration of the surveillance period. Causality determination was made using written definitions of *Related*, *Potentially Related*, *Unlikely Related*, and *Unable to Determine* (Table 1). All panel members reviewed each case independently and determined causality using these definitions. Individual panel determinations were then shared during consensus meetings either by teleconference or in-person. If panel member determinations differed, discussion of the case details occurred until panel members agreed on the determination. Majority vote was used to make final determinations if consensus could not be reached.

A Kappa score was calculated to assess intra-rater reliability of the Panel determinations of causality. A random selection of 10% of cases from each quarter was reviewed by the Panel again the following quarter to evaluate consistency. The Panel was unaware which cases were included nor were they advised of their initial decisions. The initial and secondary decisions were compared to calculate the Kappa score.

Descriptive statistics were used to characterize the relevance and evaluability of data obtained from each data source. Each data source was evaluated by the proportion of detected cases determined to be eligible and the proportion of eligible cases determined to be evaluable. A detected case was defined as any case that was identified using the search criteria specific to each data source. An eligible case was defined as a case that met inclusion criteria and indicated the relevance of each data source in relation to the patient population and case type of interest. An *evaluable* case was defined as one in which the Panel was able to determine the causal relationship of the adverse events to the reported exposure with the available information. Essentially this was any case for which the Panel determined the causal relationship was related, potentially related, or unlikely related to CCM exposure. If the causal relationship was unable to be determined then it was considered unevaluable. The evaluable cases were compared to the unevaluable cases for demographic and exposure characteristics using chi-squared tests for binary outcomes and Wilcoxon tests for non-binary outcomes.

The study was determined to be non-human subjects research by the Colorado Multiple Institutional Review Board and the consent process was not applicable.

## Results

**Fig. 1:**
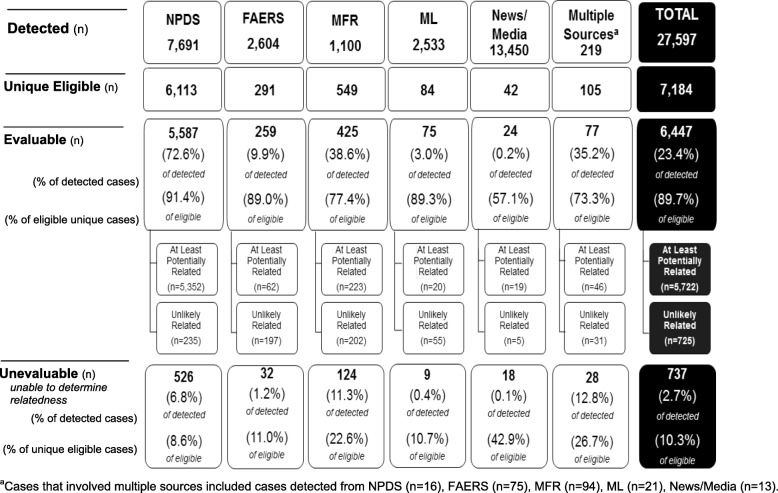
Detected, Unique Eligible, and Evaluable Cases by Data Source

A total of 27,597 reports of potential cases were detected and reviewed for program eligibility from January 2008 through September 2016. A total of 18,787 did not meet program eligibility with the four most common reasons in order of descending frequency being: report did not include an actual exposure in 11,413 (60.7%), age ≥ 12 years of age in 1834 (9.8%), foreign report in 1493 (7.9%), and no index drug exposure in 1478 (7.9%). Of the 8810 (31.9%) that met the case inclusion criteria and were deemed eligible, 7184 unique cases (7012 non-fatal and 172 fatal) were identified after duplicate cases were combined. The majority (98.5%; *n* = 7079) were sole source cases (the case was only detected within one data source). The remaining cases (1.5%; *n* = 105), included information from more than one data source with 97 of these involving 2 data sources. Most (89.5%; *n* = 94) of the multiple source cases included a manufacturer report and were commonly detected in combination with a FAERS report (56.2%; *n* = 59), a medical literature report (11.4%; *n* = 12), or a news/media report (7.6%; *n* = 8). Though the news/media data source had the highest volume of detected cases, only 0.3% (*n* = 42) of those cases were eligible for panel review and only 0.2% (*n* = 24) were evaluable. The data source with the highest likelihood of returning eligible and evaluable cases was NPDS with 7691 detected cases, 6113 (79.5%) eligible cases, and 5587 (72.6%) evaluable cases (Fig. 1).

Overall, 6447 cases were evaluable which was 23.4% of all detected and 89.7% of all eligible cases (Fig. 1). The other 10.3% (737 cases; 670 non-fatal and 67 fatal) did not contain enough information to determine causality and were judged unable to determine (unevaluable; Fig. 1; Table 2). Of the 6447 evaluable cases, 5722 (5646 non-fatal and 76 fatal) were judged as either *related* or *potentially related* to an index drug (combined and reported as cases “at least potentially related”). The remaining 725 (696 non-fatal and 29 fatal) cases were determined to be *unlikely related* to an index drug, specifying alternative causes such as underlying illnesses or unrelated conditions (e.g. meningitis, fungal pneumonia, sepsis), non-index drugs (e.g. aspirin, opioid, amoxicillin), or trauma.

**Table 2:** Eligibility and Evaluability of Non-Fatal and Fatal Cases

	NPDS (n%)	FAERS (n%)	MFR (n%)	ML (n%)	News/Media (n%)	Multiple Source^a^ (n%)	Total (n%)
Non-Fatal Unique Eligible Cases (n)	6094	272	497	78	10	61	7,012
Unevaluable (Unable to Determine Relatedness)	518 (8.5%)	27 (9.9%)	99 (19.9%)	7 (9.0%)	6 (60.0%)	13 (21.3%)	670 (9.6%)
Evaluable	5576 (91.5%)	245 (90.1%)	398 (80.1%)	71 (91.0%)	4 (40.0%)	48 (78.7%)	6342 (90.4%)
*At Least Potentially Related*	5345 (95.9%)	55 (22.4%)	204 (51.3%)	18 (25.4%)	2 (50.0%)	22 (45.8%)	5646 (89.0%)
*Unlikely Related*	231 (4.1%)	190 (77.6%)	194 (48.7%)	53 (74.6%)	2 (50.0%)	26 (54.2%)	696 (11.0%)
Fatal Unique Eligible Cases (n)	19	19	52	6	32	44	172
Unevaluable (Unable to Determine Relatedness)	8 (42.1%)	5 (26.3%)	25 (48.1%)	2 (33.3%)	12 (37.5%)	15 (34.1%)	67 (39.0%)
Evaluable	11 (57.9%)	14 (73.7%)	27 (51.9%)	4 (66.7%)	20 (62.5%)	29 (65.9%)	105 (61.0%)
*At Least Potentially Related*	7 (63.6%)	7 (50.0%)	19 (70.4%)	2 (50.0%)	17 (85.0%)	24 (82.8%)	76 (72.4%)
*Unlikely Related*	4 (36.4%)	7 (50.0%)	8 (29.6%)	2 (50.0%)	3 (15.0%)	5 (17.2%)	29 (27.6%)

^a^Case reports from more than one data source are combined to a unique case and are designated as Multiple Source

Of the 7079 eligible sole source cases, 6370 (90.0%) contained adequate information to evaluate a causal relationship between the drug exposure and reported adverse event (Fig. 1). Of the 105 eligible multiple source cases, 77 (73.3%) included adequate information for causal relationship evaluation, suggesting that data from multiple sources does not necessarily provide for a higher likelihood of evaluability (Fig. 1). However, fatal reports with more than one data source were more likely to be evaluable (Table 2).

The comparison of case characteristics between the evaluable and unevaluable cases suggests that these two groups do not differ in age or gender (*p*-values ranging from 0.193 to 0.942), or for number of adverse events reported in non-fatal cases (*p* = 0.252)(Table 3). One potential factor that could influence evaluability was the number of products as non-fatal cases were significantly (*p* < 0.001) more likely to be deemed as unevaluable and fatal cases were significantly more likely to be deemed as evaluable (*p* = 0.002) in the presence of multiple co-ingestants. Another factor for fatal cases was the availability of an autopsy. Full or partial autopsy reports were obtained for 78 (45.3%) of the 172 eligible fatal cases, 64 (61.0%) of the 105 evaluable cases, and 14 (20.9%) of the 67 unevaluable cases. Fatality cases with autopsy reports available to the Panel during case review were over twice as likely to be evaluable as those without autopsy reports (*p* = < 0.001)(Table 3).

**Table 3:** Comparison of Evaluable and Unevaluable Cases by Data Source

	NPDS		FAERS		MFR		ML		News/Media		Multiple Source		Total^1^	
Evaluable	YES	NO	YES	NO	YES	NO	YES	NO	YES	NO	YES	NO	YES	NO
Non-Fatal Cases (n)	5,576	518	245	27	398	99	71	7	4	6	48	13	6,342	670
Age (years)														
Mean (sd)	3.8 (2.6)	4.2 (3.0)	5.8 (3.3)	4.9 (2.6)	4.9 (2.9)	5.0 (2.9)	4.3 (3.4)	4.0 (3.6)	2.9 (2.3)	5.8 (3.8)	5.3 (3.1)	3.5 (2.4)	4.0 (2.7)	4.3 (3.0)
Median (min, max)	3 (0, 12)	3 (0, 11)	6 (0, 12)	4 (0, 10)	4 (0, 11)	5 (0, 11)	3 (0, 11)	3 (1, 9)	3 (1, 6)	5 (2, 11)	5 (1, 11)	4 (0, 7)	3 (0, 12)	3 (0, 11)
Gender (% male)	54.0%	51.7%	60.1%	48.1%	56.3%	56.0%	62.5%	50.0%	75.0%	40.0%	63.8%	38.5%	54.5%	51.8%
# of Products														
Mean (sd)	1.3 (0.7)	2.8 (2.8)	6.2 (7.8)	1.9 (2.5)	1.5 (1.0)	1.6 (1.3)	3.1 (3.1)	1.9 (2.3)	2.0 (2.0)	1.7 (0.5)	2.7 (2.7)	1.8 (1.1)	*1.5 (2.0)*	*2.6 (2.6)*
Median (min, max)	1 (1, 13)	2 (1, 27)	3 (1, 52)	1 (1, 14)	1 (1, 9)	1 (1, 8)	2 (1, 15)	1 (1, 7)	1 (1, 5)	2 (1, 2)	2 (1, 15)	1 (1, 4)	1 (1, 52)	2 (1, 27)
# of Index Products														
Mean (sd)	1.1 (0.3)	1.2 (0.6)	1.1 (0.3)	1.1 (0.4)	1.2 (0.4)	1.1 (0.4)	1.0 (0.2)	1.0 (0.0)	2.0 (2.0)	1.0 (0.0)	1.1 (0.2)	1.1 (0.3)	*1.1 (0.3)*	*1.2 (0.5)*
Median (min, max)	1 (1, 4)	1 (1, 7)	1 (1, 2)	1 (1, 3)	1 (1, 4)	1 (1, 4)	1 (1, 2)	1 (1, 1)	1 (1, 5)	1 (1, 1)	1 (1, 2)	1 (1, 2)	1 (1, 5)	1 (1, 7)
# of Adverse Events														
Mean (sd)	4.4 (3.0)	5.3 (4.1)	5.9 (5.0)	2.8 (2.7)	3.9 (2.8)	4.0 (3.3)	5.9 (5.7)	3.4 (6.0)	1.5 (0.6)	3.5 (1.8)	9.8 (9.4)	4.0 (1.9)	4.5 (3.3)	4.9 (4.0)
Median (min, max)	4 (1, 34)	4 (1, 35)	4 (1, 31)	2 (1, 14)	3 (1, 22)	3 (1, 19)	4 (1, 29)	1 (1, 17)	2 (1, 2)	4 (1, 6)	6 (1, 38)	3 (2, 7)	4 (1, 38)	4 (1, 35)
Fatal Cases (n)	11	8	14	5	27	25	4	2	20	12	29	15	105	67
Age (years)														
Mean (sd)	2.6 (3.4)	2.0 (2.0)	4.0 (4.2)	1.7 (1.7)	1.1 (1.5)	1.7 (2.4)	2.6 (3.9)	0.8 (0.5)	2.8 (3.4)	3.3 (3.3)	1.5 (1.7)	1.1 (1.1)	2.1 (2.9)	1.9 (2.3)
Median (min, max)	1 (0, 11)	2 (0, 5)	2 (0, 11)	1 (0, 4)	1 (0, 7)	1 (0, 8)	1 (0, 7)	1 (1, 1)	1 (0, 11)	3 (0, 11)	1 (0, 5)	1 (0, 4)	1 (0, 11)	1 (0, 11)
Gender (% male)	45.5%	50.0%	75.0%	75.0%	48.0%	57.9%	66.7%	50.0%	65.0%	36.4%	62.1%	73.3%	59.0%	57.6%
# of Products														
Mean (sd)	2.1 (0.7)	2.0 (1.2)	11.3 (15.5)	2.6 (2.5)	2.3 (1.8)	2.1 (2.0)	4.3 (2.5)	3.0 (2.8)	2.0 (1.2)	1.6 (1.2)	2.7 (1.9)	2.8 (1.9)	*3.6 (6.5)*	*2.2 (1.8)*
Median (min, max)	2 (1, 3)	2 (1, 4)	4 (1, 44)	2 (1, 7)	2 (1, 7)	1 (1, 10)	5 (1, 7)	3 (1, 5)	2 (1, 5)	1 (1, 5)	2 (1, 9)	2 (1, 6)	2 (1, 44)	1 (1, 10)
# of Index Products														
Mean (sd)	1.1 (0.3)	1.1 (0.4)	1.4 (0.9)	1.2 (0.4)	1.5 (1.0)	1.2 (0.6)	1.8 (1.5)	1.0 (0.0)	1.1 (0.3)	1.1 (0.3)	1.6 (0.7)	1.7 (1.0)	1.4 (0.8)	1.3 (0.7)
Median (min, max)	1 (1, 2)	1 (1, 2)	1 (1, 4)	1 (1, 2)	1 (1, 5)	1 (1, 4)	1 (1, 4)	1 (1, 1)	1 (1, 2)	1 (1, 2)	1 (1, 3)	1 (1, 4)	1 (1, 5)	1 (1, 4)
Autopsy Available	7 (63.6%)	3 (37.5%)	3 (21.4%)	1 (20.0%)	16 (59.3%)	2 (8.0%)	1 (25.0%)	0 (0.0%)	15 (75.0%)	0 (0.0%)	22 (75.9%)	8 (53.3%)	*64 (61.0%)*	*14 (20.9%)*

^1^Italicized numbers indicate statistical significance between Total Evaluable and Total Unevaluable at p<0

The strengths and limitations of each data source are summarized in Table 4. Limitations common to all data systems include that not all cases are reported and self-reported data is relied upon in 4 of 5 data sources (NPDS, FAERS, participating manufacturer postmarket safety databases, and news/media reports). NPDS, FAERS, and participating manufacturer postmarket safety databases have common strengths of structured data collection. Each data source contributes unique strengths, reinforcing the mosaic approach.

**Table 4:** Strengths and Limitations of Pediatric Cough and Cold Safety Surveillance System Data Sources

	Strengths	Limitations
National Poison Data System (NPDS)	• Structured data collection forms with required fields and common product database • National coverage • Medical management cases including follow-up and disposition when possible, often with healthcare providers • Timely availability of data • Legacy data since 1983	• Not all exposures are reported • Self-reported exposures; most from non-clinical reporters
FDA Adverse Event Reporting System (FAERS)	• Structured data collection forms • Publicly accessible datasets • Regulated reporting requirements • Legacy data since 1998	• Spontaneous reports; not all cases are reported • Changes in reporting requirements over time • Inconsistencies in reporting practices by manufacturers • Self-reported cases; many from non-clinical reporters • Duplicate cases • Incomplete reports • Delays in public data access
English Language Medical Literature	• Peer-reviewed publication • Detailed clinical course	• Unstructured data • Not all cases are reported • Reporter bias (author and journal editors determine what may be of interest to report)
Participating Manufacturer Postmarket Safety Databases	• Structured data collection forms • Follow-up and disposition documented when possible, often with healthcare providers	• Spontaneous reports; not all cases are reported • Self-reported cases; many from non-clinical reporters • Inconsistencies in documentation practices by manufacturers
News/media Reports	• Often include contextual information about exposures • Higher likelihood of detecting fatal events • Timely reporting	• Unstructured data • Not all cases are reported • Reporter bias (journalist determines what is of interest to report) • Non-clinical reporters

## Discussion

Each data source utilized to evaluate the safety profile of pediatric CCM exposures yielded variable detection, eligibility, and evaluability rates, with NPDS yielding the highest and news/media yielding the lowest proportion of eligible and evaluable cases. The overall data system with cases systematically gathered from NPDS, FAERS, English-language medical literature, manufacturer postmarket safety databases, and news/media reports provided a comprehensive look at exposures that otherwise cannot be studied in clinical trials. Once eligible cases were identified, 9 out of 10 were evaluable. The Code of Federal Regulations (21 CFR 314.80; 21 CFR 314.98) specifies the four data elements needed for adverse event reporting: identifiable patient, identifiable reporter, suspect drug or biological product, and an adverse event or fatal outcome [12]. While all data sources utilized can provide these four elements in some format, they vary greatly in the completeness and value of the information.

FAERS (formerly known as AERS) system has been available since 1998 and is intended to support the FDA’s postmarketing safety surveillance program for drug and therapeutic biologic products. This is the primary data source used by the FDA to monitor the safety of regulated products. Quarterly reports are generated to call attention to products or drug substances that have safety signals. Healthcare professionals and patients can submit voluntary reports while sponsors have mandatory reporting for specific types of cases (sponsors may also submit voluntary reports). The mandatory reporting requirements have changed over the years, specifically OTC medications marketed under the monograph system. Prior to the Dietary Supplement and Nonprescription Drug Consumer Protection Act signed in 2006 by Congress, there were no mandatory reporting requirements of sponsors for OTC medications not marketed under an application and all reporting was voluntary. The Act became effective in December 2009, requiring sponsors to report serious adverse events involving OTC medications. This reporting requirement alone must be considered when evaluating data from that source in that the majority of events in the system are expected to be serious and that trends over time should not be considered prior to the 2009 effective date. However, sponsors vary in submission of voluntary reports, so some trends may be related to internal policies rather than true signals. Incomplete reports diminish the value of this otherwise useful system as well as high number of duplicate reports. In this study, missing data related to suspect products (including specific active ingredients), dose involved, and clinical course (timing of exposure, treatments, and adverse events) greatly impacted the evaluability of cases. Reporting rates calculated strictly from FAERS without manual review is concerning considering only 1 in 10 of detected cases were evaluable. While FAERS would be expected to be the primary source for FDA regulated product safety data, concerns of completeness, timeliness, and accurate data were noted following an investigation by the Government Accountability Office [13]. The criticisms identified are not insurmountable and could be addressed with improvements in the collection system and requirements, utilizing innovative ways to identify duplicates and reduce incomplete submissions. Despite these challenges, the strengths of FAERS, including structured data collection forms (paper and electronic), publicly accessible datasets, and regulated reporting requirements, make this a valuable postmarket surveillance data source.

NPDS data have been used for decades to evaluate the safety profile of countless pharmaceutical and non-pharmaceutical products as well as an early detection system for emerging public health concerns [14–19]. Legacy data is available as far back as 1983. The primary purpose of poison centers is to provide round-the-clock medical management by healthcare professionals free of charge to the public and other healthcare providers. Since the primary purpose is medical management, follow-up is conducted as part of standard practice and recorded in the database. Every exposure is captured in the NPDS in real-time (case records uploaded to a national repository every few minutes). A common product database (Micromedex® POISINDEX®, Truven Health Analytics) ensures standardized product identification and allows for product-specific analysis. These structured data, captured electronically with required key data fields, allow for identification of relevant cases and inclusion of critical information needed to evaluate them as NPDS yielded the highest rate of both unique eligible and evaluable cases (3 in 4 detected cases were evaluable). However, not all exposures are reported to NPDS and certain types of events, such as pediatric accidental unsupervised ingestions, are more likely to be reported than others like minor adverse events with appropriate use. Reporting bias of fatalities is also likely. Unfortunately no national death database adequately records specific products involved or systematically evaluates causality, particularly no generally accepted practice for obtaining toxicology as part of autopsy. While poison centers do not capture all deaths, trends are comparable to national mortality data captured by the National Vital Statistics System [20, 21].

Manufacturer reports are similar to both FAERS and NPDS in that they do allow for direct communication with healthcare providers to collect and record the information but must rely on spontaneous reports. Most manufacturer systems will use data collection tools similar to FAERS considering certain cases will need to be submitted in standardized form (e.g., MedWatch forms). However, these forms still allow for missing data similar to those already noted for FAERS which impact the evaluability of cases. This source yielded the highest number of fatal cases but a moderate rate of eligibility and evaluability with approximately 1 evaluable case for every 3 detected. Perhaps the greatest value of this data source is the capture of cases that do not meet FAERS mandatory reporting requirements.

Medical literature and news/media reports introduced a different reporting bias which is that of the reporter rather than the patient or medical provider. The author or media outlet determine what cases they publish. Publishing decisions can be driven by political issues, personal or professional interests, emerging public health issues, new sentinel events, and current events. These outlets can provide detailed contextual information on cases that would not otherwise be detected (e.g., fatalities), however the information is often relayed by a lay person and not translational, complete, accurate, or supported by any source documentation. These reports can also be influenced by lawyers involved in medical legal cases. The speculative, and sometimes theatrical, reports from media can introduce significant bias and contribute to the background noise in FAERS considering significant events will have to be reported regardless of the completeness or accuracy of the report and once submitted are not typically retracted even if it was determined to be a false report. Medical literature and news/media reports yielded the lowest overall rates of eligibility and evaluability (1 evaluable for every 34 detected for literature; 1 in 500 for news/media) which highlight the importance of determining the data sources most relevant to the surveillance objectives. However, when cases are stratified by outcome, news/media was the only source with more unique eligible fatal cases (*n* = 32) than non-fatal cases (*n* = 10). Evaluability of the fatal cases was similar to that of other data sources, suggesting news/media’s strengths could be in identifying serious events not otherwise detected. Weighing the incremental benefit of a few new potentially biased cases with the cost of continual monitoring of a large amount of information and in mitigating false reports is important.

Another challenge with postmarket surveillance is the representativeness of the reports obtained. Overall there did not appear to be a differential bias between the evaluable and unevaluable cases, suggesting generalizability of study results. While this is true for pediatric CCM surveillance it may not hold true for other evaluations. Case specifications (inclusion criteria) must be matched with the most relevant data sources for the exposure type, population, and behaviors of interest. Hence, a limitation of this study is that the quality and value of these data sources may vary when employed to address safety profiles of other drug classes or products. The same limitation is true for generalizability of Panel intra-rater reliability. The CCM Panel membership was the same throughout the study period and reliability estimates indicated consistency over time. Panel determinations of relatedness yielded a kappa of 0.790 (95% confidence limit 0.74, 0.85) which is considered substantial agreement. [22] While Panel reliability was high for CCM evaluations, the level of intra-rater reliability may vary in other studies depending on the key outcome measures and definitions, availability of information, and ability to create a consistent review process to gain consensus.

## Conclusion

Understanding the detection rate, eligible case rate, and evaluable case rate for each data source is informative when evaluating the appropriateness and quality of these data sources. The data sources employed in the Pediatric Cough and Cold Safety Surveillance System were deemed valuable. Each data source contributed unique cases although the resources required for the sources with low-yield evaluable cases warrants consideration. While this study illustrates the value of each data source and the generalizability of the results, improvements are needed in case completeness and accuracy to enhance the quality of postmarket safety evaluations.

## Additional file

Additional file 1: The additional file is an appendix (Title: Methods Appendix) that contains the case inclusion criteria, data source specifications, and case processing flow diagram. (DOC 406 kb)

## Abbreviations

AAPCC: American Association of Poison Control Centers
API: active pharmaceutical ingredient
CCM: cough cold medication
FAERS: FDA Adverse Event Reporting System
FDA: United States Food and Drug Administration
ICH: International Conference of Harmonisation
ICSR: Individual Case Safety Report
MedDRA: Medical Dictionary for Regulatory Activities
NPDS: National Poison Data System
OTC: over-the-counter
PC: Poison Center
