# Supplementary material for: Evaluation of the quality and value of data sources for postmarket surveillance of the safety of cough and cold medications in children
Source: BMC Med Res Methodol. 2018 Dec 22;18:175. doi: 10.1186/s12874-018-0626-3 (PMC6303866; doi:10.1186/s12874-018-0626-3)
Supplement: Supplementary file 1 — The additional file is an appendix (Title: Methods Appendix) that contains the case inclusion criteria, data source specifications, and case processing flow diagram. (DOC 406 kb) [file 12874_2018_626_MOESM1_ESM.doc]

**Appendix. Case Inclusion Criteria, Data Source Specifications, and Case Processing Flow**

| **Case Inclusion Criteria** |
| --- |
| - Age <12 years - Exposure to at least one of 8 index ingredients: brompheniramine, chlorpheniramine, dextromethorphan, diphenhydramine, doxylamine, guaifenesin, phenylephrine, pseudoephedrine - Ingestion of an oral index product - Report of at least one serious adverse event as defined by Data Source Inclusion Criteria - Exposure occurred in the United States - Case detected between 01 January 2008 and 31 December 2016 with event occurring prior to 30 September 2016 |

| **Data Source Specifications: National Poison Data System (NPDS)** |
| --- |
| NPDS is the data repository of exposure records reported to United States Regional Poison Centers (RPCs). Data for exposures reported to the NPDS were collected at participating poison centers and delivered to the Rocky Mountain Poison & Drug Center (RMPDC). As of study closure, 51of 55 RPCs were participating in data collection and 6,466 of 6,752 eligible cases detected at individual poison centers were submitted to RMPDC. Upon submission from the RPCs, cases were processed to confirm study eligibility and to identify duplicates. |
| *Case Detection Criteria*   - All exposure reasons - Patient age:   Actual Age: <12  Estimated Age: ≤5 years   - Call type: Exposure - Case status: Closed - Medical Outcome: Moderate effect, major effect, or death - Product code contained one or more of 8 index ingredients - Case detection date: 01 January 2008 to 31 March 2017 |

| Data Source Specifications: FDA Adverse Event Reporting System (FAERS) |
| --- |
| FAERS is a database that contains adverse event reports, mediation error reports, and product quality complaints that result in adverse events submitted to the FDA. FAERS data files are posted quarterly and include patient-level reports. These files are publically available and typically posted on the FDA website approximately 3-9 months after the close of a calendar quarter. Once posted, available data fields were searched to identify potentially eligible cases per the criteria listed below. Full reports of cases meeting selection criteria were then requested from the FDA via the Freedom of Information (FOI) Act. Upon submission from the FDA, cases were processed to confirm study eligibility and to identify duplicates. |
| *Case Detection Criteria*   - Child <12 years of age - Case involved a product containing one or more of 8 index ingredients or known cough/cold product name - FDA received date: 01 January 2008 to 31 March 2017 |

| **Data Source Specifications: Medical Literature** |
| --- |
| Using PubMed, Ovid MEDLINE (Ovid MEDLINE(R) In-Process & Other Non-Indexed Citations, Ovid MEDLINE(R) Daily and Ovid MEDLINE(R) <1950 to Present>), and EMBASE databases, the English language medical literature was searched weekly for records using the criteria listed below. All resultant abstracts were screened and the full text for pediatric human exposures to a product containing one or more of 8 index ingredients were obtained. Full text articles were then reviewed for program eligibility and to identify duplicates. |
| *Case Detection Criteria*   - Search terms:   - brompheniramine   - chlorpheniramine   - dextromethorphan   - diphenhydramine   - doxylamine   - guaifenesin   - phenylephrine   - pseudoephedrine - Language: English - Any adverse event - Publication date: 01 January 2008 to 31 March 2017 |

| **Data Source Specifications: Participating Manufacturer Postmarket Safety Databases** |
| --- |
| Eight participating manufacturers searched their internal safety databases for eligible cases using the selection inclusion criteria listed below. Cases were de-identified (e.g., removal of birthdate or name) by the manufacturer and submitted to RMPDC on a quarterly basis, at which time they were reviewed for completeness and eligibility. |
| *Case Detection Criteria*   - Closed cases (cases which have completed the review process by the manufacturer or have received updates since the previous quarter) - Child <12 years of age - Suspect product includes 1 or more of 8 index ingredients - Adverse event judged as serious according to International Conference of Harmonization (ICH) definition of serious adverse event - Manufacturer received date: 01 January 2008 to 31 March 2017 |

| **Data Source Specifications: News/media Reports** |
| --- |
| Using an online tool (DJX, Dow Jones, New York, New York) that aggregates media/news reports, indexed reports were searched twice a quarter for mentions using the search criteria listed below. The full-text version of each report headline was then reviewed for program eligibility. |
| *Case Detection Criteria*   - Search terms:   - brompheniramine   - chlorpheniramine   - dextromethorphan   - diphenhydramine   - doxylamine   - guaifenesin   - phenylephrine   - pseudoephedrine   - child   - children   - kid   - baby   - infant   - pediatric   - toddler   - pediatric cough and cold - Language: English - Any adverse event - Report date: 01 January 2008 to 31 March 2017 |

**Case Processing Flow**
